# Intermanual transfer and retention of visuomotor adaptation to a large visuomotor distortion are driven by explicit processes

**DOI:** 10.1371/journal.pone.0245184

**Published:** 2021-01-11

**Authors:** Jean-Michel Bouchard, Erin K. Cressman

**Affiliations:** School of Human Kinetics, University of Ottawa, Ottawa, Canada; West Virginia University, UNITED STATES

## Abstract

Reaching with a visuomotor distortion in a virtual environment leads to reach adaptation in the trained hand, and in the untrained hand. In the current study we asked if reach adaptation in the untrained (right) hand is due to transfer of explicit adaptation (EA; strategic changes in reaches) and/or implicit adaptation (IA; unconscious changes in reaches) from the trained (left) hand, and if this transfer changes depending on instructions provided. We further asked if EA and IA are retained in both the trained and untrained hands. Participants (n = 60) were divided into 3 groups (Instructed (provided with instructions on how to counteract the visuomotor distortion), Non-Instructed (no instructions provided), and Control (EA not assessed)). EA and IA were assessed in both the trained and untrained hands immediately following rotated reach training with a 40° visuomotor distortion, and again 24 hours later by having participants reach in the absence of cursor feedback. Participants were to reach (1) so that the *cursor* landed on the target (EA + IA), and (2) so that their *hand* landed on the target (IA). Results revealed that, while initial EA observed in the trained hand was greater for the Instructed versus Non-Instructed group, the full extent of EA transferred between hands for both groups and was retained across days. IA observed in the trained hand was greatest in the Non-Instructed group. However, IA did not significantly transfer between hands for any of the three groups. Limited retention of IA was observed in the trained hand. Together, these results suggest that while initial EA and IA in the trained hand are dependent on instructions provided, transfer and retention of visuomotor adaptation to a large visuomotor distortion are driven almost exclusively by EA.

## Introduction

Reaching with altered visual feedback of the hand’s position in a virtual reality environment leads to motor adaptation in the trained hand and also in the untrained hand [1–4]. The phenomenon of intermanual transfer of visuomotor adaptation has been studied in the lab by having participants reach to visual targets with a single hand while seeing a cursor on a screen that is misplaced relative to their actual hand position (e.g., the cursor’s trajectory is rotated relative to actual hand motion). Following these reach training trials with the trained hand, participants are asked to reach to targets with the opposite (untrained) hand in the presence [2, 5–7] or absence of the visuomotor distortion [3, 8–10].

Initial findings led to the proposal that intermanual transfer of visuomotor adaption arises because the underlying processes are effector independent [see 11]. Specifically, both hands have shared access to a sensorimotor map that has been updated implicitly (i.e., unconsciously) through error-based motor learning, such that motor commands have been adapted to minimize the difference between predicated and actual sensory feedback experienced [12]. This explanation for intermanual transfer of visuomotor adaptation has recently been called into question given that the extent of transfer has been shown to vary depending on experimental design. For example, the extent of transfer has been shown to differ depending on which hand is trained first [2, 8, 10], the location of the targets in the workspace [6], and the visibility of the cursor during reaches with the untrained limb [2, 3, 9]. Together, these results suggest that the two hands do not have the same access to the new sensorimotor mapping and/or that additional learning processes [see 13] may differentially contribute to visuomotor adaptation in the trained and untrained hands.

In accordance with this latter suggestion, it has recently been suggested that explicit processes play a role in visuomotor adaptation [14–18], as well as the retention [19–21] and transfer of visuomotor adaptation between limbs [22–24]. The processes encompassed by the term *explicit* vary within the literature, depending on manner of assessment. Taylor and colleagues [17] refer to explicit processes as reflecting pre-planned aiming strategies, while Werner and colleagues [25] refer to explicit knowledge as reflecting awareness of the nature of the perturbation as demonstrated by the use of cognitive strategies to counteract the visuomotor distortion [see 7, 15]. In the current study explicit processes are defined in accordance with Werner and colleagues’ [25] definition, such that explicit processes reflect the engagement of cognitive strategies in order to counteract a given visuomotor distortion, where these strategies can arise due to instructions provided, or on the participant’s own initiative. In contrast, we define implicit processes as reflecting changes in participants’ reaches that arise in the absence of their awareness of these changes. This implicit adaptation is driven by error-based motor adaptation as described above, in response to a sensory prediction error [12].

Preliminary research looking to establish the role of explicit processes in intermanual transfer did so indirectly. Specifically, experimenters looked to manipulate participants’ awareness of a visuomotor distortion by having them reach with a small cursor rotation (between 22.5° - 32°), that was introduced gradually or abruptly [7, 9]. Researchers assumed that gradually introducing the cursor rotation by small increments over trials would limit participants’ awareness of the visuomotor distortion (and hence engagement of explicit processes), while introducing the cursor rotation abruptly (i.e., all at once) would lead to participants being more aware of the visuomotor distortion (and hence lead to the engagement of a cognitive strategy). To further manipulate participants’ level of awareness of the visuomotor distortion, Wang and colleagues [7] also included a third group of participants who were told about the cursor rotation (i.e., told that the cursor would be rotated 32° CCW relative to hand motion). Using this methodology, both Wang et al. [7] and Taylor et al. [9] found that the extent of intermanual transfer was similar across groups of participants, even participants who were instructed on the nature of the visuomotor distortion. Based on these results, the authors concluded that awareness of the visuomotor distortion, and hence explicit processes engaged, have little effect on intermanual transfer. Instead, implicit processes underlie intermanual transfer of visuomotor adaptation.

Critically, it is unclear if manipulating the introduction of the cursor rotation (gradual vs. abrupt vs. instructed introduction), led to groups of participants differentially engaging in explicit and implicit processes. Debriefing reports imply that some of the participants assigned to the gradual groups in the work by Wang et al. [7] and Taylor et al. [9] were aware of changes in the visuomotor environment, and hence may have engaged in a strategy. Alternatively, recent work has demonstrated that small cursor rotations (i.e., less than 40°) lead to limited engagement of explicit processes, even when the visuomotor distortion is explained to participants in advance [18, 25]. Thus, awareness of the distortion, and hence engagement of cognitive strategies to counteract a given visuomotor distortion, may have been more similar across groups than expected, making it difficult to establish the role of explicit and implicit processes in intermanual transfer of visuomotor adaptation. It is also important to note that assessment trials used to establish intermanual transfer did not separate the potential contributions of explicit and implicit processes, giving rise to the possibility that the contribution of explicit and implicit processes could have differed across groups but led to similarities in the amount of intermanual transfer observed.

Recently, Werner and colleagues adopted the process dissociation procedure (PDP) in attempt to explore the role of explicit processes in intermanual transfer more directly [24]. The PDP originally put forth by Jacoby [26], is widely used in cognitive psychology and is founded on the premise that conscious knowledge is controllable. In other words, changes in reaches that arise explicitly due to participants engaging in a cognitive strategy can be dissociated from implicit changes in reaches, by asking participants to express or repress a learned behaviour respectively [24, 25]. In Werner et al. [24], participants trained with a small (30°) or large (75°) cursor rotation, that was introduced gradually (G) or abruptly (A). Werner found that participants who trained with the abruptly introduced large cursor rotation (i.e., A75°) showed greater explicit changes (i.e., adaptation) in the trained hand compared to the other groups. More importantly, they also demonstrated greater intermanual transfer, implying that intermanual transfer is directly related to the extent of explicit adaptation in the trained hand.

In the current research we looked to extend Werner’s findings by asking if explicit and/or implicit adaptation in the trained (left) hand transfer to the untrained (right) hand. We further asked if the contributions of explicit and implicit adaptation to intermanual transfer change depending on instructions provided to participants, and hence how they develop cognitive strategies to counteract the visuomotor distortion. We have recently shown that the contributions of explicit and implicit processes to visuomotor adaptation in the trained hand are modified depending on whether participants are provided with a cognitive strategy on how to counteract the visuomotor distortion or must develop a cognitive strategy on their own accord [18]. In order to probe the role of instructions on intermanual transfer, we adopted the PDP used by Werner and colleagues [24, 25], and assessed explicit and implicit visuomotor adaptation in the trained and untrained hands for our Instructed and Non-Instructed groups of participants. Explicit and implicit contributions to visuomotor adaptation were assessed following initial visuomotor adaptation training and again 24 hours later, in order to establish retention of explicit and implicit adaptation in the trained and untrained hands. As indicated above, evaluation of explicit and implicit adaptation within the PDP, assumes that participants can engage and disengage from using a cognitive strategy when asked to do so. Furthermore, the method assumes that visuomotor adaptation can be driven by both explicit and implicit processes (at the same time), regardless of participants’ awareness of the visuomotor distortion (i.e., awareness of the visuomotor distortion and the use of a reaching strategy are not the same).

As a final experimental manipulation, we included a third group of participants (our Control group). This group was instructed to reach so that their hand went to the target on all PDP trials, and hence these participants were never asked to express or repress a learned behaviour. The Control group allowed us to establish if the act of asking participants to engage in strategic reaching promotes explicit adaptation at the expense of implicit adaptation (e.g., as suggested by [27, 28]). We hypothesized that the Instructed group would demonstrate the greatest explicit adaptation in the trained hand. With respect to the manipulation of instructions and its influence on intermanual transfer, we expected that participants in the Instructed group would demonstrate the greatest transfer of explicit adaptation from the trained to the untrained hand, and this would be retained in both hands. Transfer of implicit adaptation was expected to be highest in the Control group, who was not instructed to express a learned behaviour at any time. Together, these results would indicate that explicit and implicit processes contribute to the intermanual transfer and retention of visuomotor adaptation, but that their contributions depend on instructions provided.

## Methods

### Participants

Sixty naïve participants, aged 19–30 years (M = 22 years, SD = 2.5), were recruited from the University of Ottawa community to participate in this study. Participants were right handed as determined by the modified version of the Edinburgh Handedness scale (M = 80, SD = 16.8; [29]) and self-reported having normal or corrected-to-normal vision, and no neurological, sensory or motor dysfunction. All participants provided written informed consent and the experiment was approved by the University of Ottawa Research Ethics Board.

Testing took place over 2 consecutive days. The first session lasted approximately 40 minutes, while the second session (taking place approximately 24 hours later) lasted 25 minutes. Participants were randomly assigned to one of three groups: i) an Instructed group (n = 20), ii) a Non-Instructed group (n = 20), or iii) a Control group (n = 20). One participant from the Instructed group was removed from the analyses and results reported below due to their reaching errors falling 3 standard deviations above the group’s mean.

### Experimental apparatus and set-up

**Fig 1:**
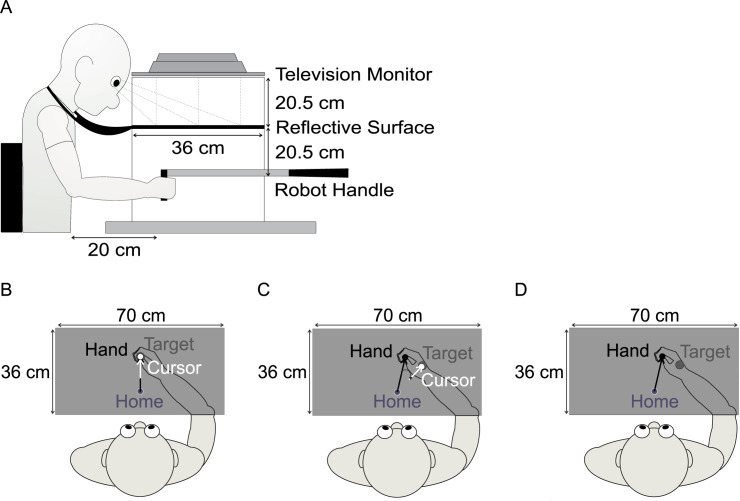
Experimental apparatus and reach conditions. (A): Side-view of the experimental apparatus. Participants were instructed to grasp the robot handle with either the left or right hand. (B): Top view of the experimental apparatus and visual feedback displayed during reach training trials with an aligned cursor. (C): Visual feedback displayed during reach training trials with a rotated cursor. (D). Example of a Catch trial or PDP trial with no visual feedback. For (B–D), the target was displayed as a green circle (shown here as a dark grey circle), and the cursor as a white circle. Thin white and black arrows represent the trajectory of the cursor movement and actual hand movement respectively (note the actual hand movement was not seen during testing).

Participants were seated comfortably in a height-adjustable chair located in front of the experimental apparatus. They were asked to grasp the handle of a two-joint robot manipulandum (KINARM; Kingston, Canada) that moved in a 70 cm by 36 cm work area. Visual targets were projected from a downward facing monitor (EzSign model 47LD452B; refresh rate 60 Hz; LG Seoul, South Korea), located 20.5 cm above a reflective surface that was located 20.5 cm above the robot handle. Thus, visual stimuli appeared to lie in the same plane as the reaching hands (Fig 1A). Participants’ view of their limbs was obstructed by a black cloth draped between their shoulders and the apparatus. The lights in the room were turned off once participants were comfortably seated and ready to begin testing.

During testing, the location of the reaching hand (left or right) holding the robot manipulandum was displayed as a white circular cursor (1 cm in diameter) on the reflective surface. In general, participants reached from a home position (1 cm in diameter, located inline with their body midline and approximately 20 cm in front their chest) to one of three visual targets, which were represented by green circles (1 cm in diameter) and located 15 cm away from the home position. The center target was straight-ahead, aligned with body midline, and the remaining two targets were positioned either 52° CW or CCW with respect to straight-ahead. The targets were presented randomly, such that each target was presented once before a target was repeated.

### Experimental procedure and trials

**Fig 2:**
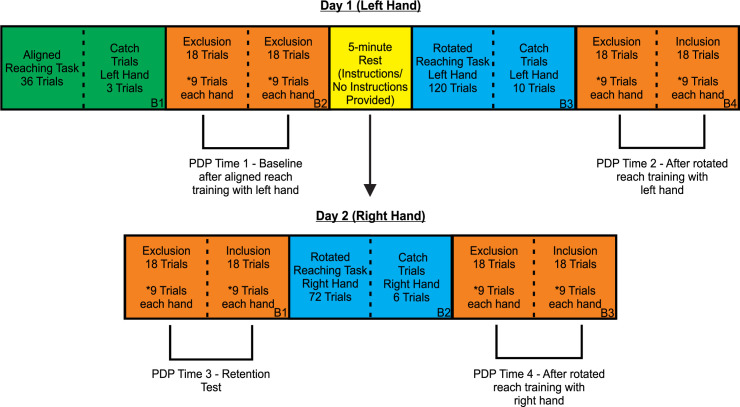
Experimental design. Outline of testing Blocks completed by all participants. All Blocks are outlined by solid black lines and the Block number is indicated in the bottom right corner. Dotted lines represent Block division by type of trial completed. The top portion indicates blocks completed on the first day of testing, while the lower portion represents blocks completed on the second testing day (24 hours later). During PDP Blocks, the order of exclusion and inclusion trials, and the hand that participants initially reached with (left vs. right hand), were counterbalanced across participants. Participants assigned to the Control group were instructed to, “reach so that your hand lands on the target” on all PDP trials.

All participants completed 4 testing blocks on Day 1, and 3 testing blocks on Day 2 (see Fig 2).

#### Reach training trials

Reach training trials in testing Blocks 1 and 3 on Day 1 were completed with the left non-dominant hand, while reach training trials in Block 2 on Day 2 were completed with the right dominant hand. In the reach training trials, participants were instructed to reach to the target as quickly and accurately as possible so that the white cursor landed on the target. Trials began with participants firmly grasping the robot manipulandum. The robot then moved the participant’s hand so that their hand and cursor were at the home position. The hand was held at the home position for 500 ms. After this time, the home position and cursor disappeared, and one of the three green targets appeared, cuing participants to start their movement. Once participants had moved 7 cm away from the home position, they saw the white cursor again, representing their current hand position. This position corresponded to the location at which peak velocity was typically achieved, hence limiting participants’ ability to make corrections early in the movement in response to online visual feedback [30].

Participants continued to reach until the cursor stopped on the green target (i.e., the center of the cursor and the center of the target were within 0.5 cm). At this time, the target disappeared, and the trial was considered complete. The participant’s hand was held at the final position for 500 ms. Following this delay, the robot passively returned the hand to the starting position (movement time = 1000 ms in duration). The robot handle followed a linear grooved path from the target position to the home position and no visual feedback was provided. If participants attempted to move outside of the linear path, a resistance force (proportional to the depth of penetration with a stiffness of 2 N/mm and a viscous dampening of 5 N/mm) perpendicular to the grooved path was produced. The position of the KINARM robot was recorded at a sampling rate of 1000 Hz, with a spatial accuracy of 0.1 mm.

In Block 1 on Day 1, participants reached with aligned visual feedback of their left hand position for 36 trials, such that the white cursor accurately represented where the hand was located in the work area (Fig 1B). During Block 3 on Day 1 (120 trials) and Block 2 on Day 2 (72 trials), participants reached to targets with misaligned visual feedback of their left and right hand positions, respectively. Specifically, the trajectory of the cursor was rotated 40° CW from a participant’s hand trajectory, such that they had to reach 40° CCW, or in a leftward direction from the target, to have the cursor accurately land on the intended target (Fig 1C). The purpose of completing reach training trials on Day 2 with the right hand was to ensure that participants demonstrated adaptation during reach training trials, and subsequent implicit adaptation (as outlined below) in the right hand. Given previous results from our lab [18], demonstrating that reach adaptation achieved an asymptote after approximately 60 reach training trials with the right hand to 3 targets in a similar set-up to the current experiment, we decreased the number of reach training trials on Day 2.

Randomly interspersed catch trials (1 for every 12 reach training trials), were included in the reach training blocks. These trials were included as a means of measuring baseline levels of performance during aligned reach training and were compared to catch trials within rotated reach training blocks to confirm that participants adapted their movements to the visuomotor distortion. These catch trials were similar to reach training trials, however, no visual feedback regarding hand position was provided at any time during the reach trajectory (Fig 1D). A catch trial was considered complete once velocity decreased below 0.01m/s and participants held their final hand position for 500 ms.

#### Process dissociation procedure (PDP)

Participants assigned to the Instructed and Non-Instructed experimental groups performed 36 total trials in each of the 4 PDP blocks. 18 of these trials were performed with the right hand and the other 18 with the left hand. The 18 trials for each hand were further divided into 9 exclusion trials and 9 inclusion trials (3 trials to each target). The order in which participants completed the exclusion and inclusion trials, as well as the hand that participants used first (i.e., left or right hand), were counterbalanced across participants. Participants performed all 18 trials of a certain type (exclusion or inclusion), 9 with each hand, before performing the second type of trials (inclusion or exclusion). In the exclusion PDP trials, participants were asked to “refrain from using a strategy or anything you have learned during reach training trials, when reaching to the targets, and reach so that your *hand* lands on the target.” In the inclusion trials, participants were instructed to “use any strategy you have developed or use anything you have learned during reach training trials, to reach to the targets, so that your *cursor* lands on the target.” Blocks of PDP trials were performed prior to the introduction of the rotation (Day 1, Block 2 (PDP Time 1) and Day 2, Block 1 (PDP Time 3)), as well as after rotated reach training on both days of testing (Day 1, Block 4 (PDP Time 2) and Day 2, Block 3 (PDP Time 3)) (Fig 2). Note that all 36 PDP trials performed at PDP Time 1, were exclusion trials, as participants had not yet reached with the visuomotor distortion. All PDP trials were performed in a similar manner as the catch trials, such that no cursor was presented.

Participants assigned to the Control group also completed 36 trials within each PDP Block, separated into 2 sets of 18 trials (i.e., 2 x (9 trials with the left hand + 9 trials with the right hand)). On all PDP trials, these participants were instructed to, “reach so that your *hand* lands on the target.”

#### Groups of participants: Instructions

Immediately following the first block of PDP trials on Day 1 (Block 2, Fig 2), participants assigned to the Instructed group were informed of the upcoming rotation and instructed on how to counteract it. Specifically, these participants were shown a modified clock face, similar to that used by Benson et al. [15], and told they needed to aim two numbers to the left of the target to compensate for the 40˚ CW rotation. Participants demonstrated their understanding of the nature of the rotation and the instructions provided to them by drawing an arrow towards where they needed to reach to counteract the visuomotor distortion for one of the three targets. Participants were reminded of the instructions halfway through the rotated reach training trials on Day 1 of testing (i.e., after 65 trials). They were not reminded of the instructions on Day 2. Participants in the Non-Instructed and Control groups were not provided with instructions, nor told that a visuomotor distortion would be introduced. Instead, they sat quietly for approximately 5 minutes after Block 1 on Day 1, before testing resumed.

#### Post-experiment questionnaire

After having completed testing on Day 2, all participants were asked a series of questions to assess their verbal awareness of the visuomotor distortion at the end of the experiment. Participants were asked about the reach training trials completed with their left and right hands. The questions asked were the same as those used by Benson et al. [15], such that participants were classified as being aware of the visual distortion if they noticed a change in how they had to reach to get the cursor to the intended targets (i.e., the cursor did not go where they intended it to), or reported using a strategy (e.g., reaching to the left of the target). If participants indicated that they did not notice the task change in any way, or provided no indication of being aware that a cursor rotation/distortion had been presented they were classified as unaware.

### Data analyses

All reaching trials (reach training and PDP trials) were visually screened and analyzed using custom written MATLAB programs. The start and end points of each trial were selected based on a velocity criterion, such that the start and end points were defined as when velocity first increased above or decreased below 0.01m/s for a minimum of 50 ms, respectively. For each trial, reaction time (RT), movement time (MT), and angular error at peak-velocity (PV) were determined, such that the angular error was defined as the difference between a vector joining the home position to the desired target, and a vector joining the home position to the hand’s location at peak velocity.

We first looked to establish if participants adapted to the 40° cursor rotation on Day 1 with the left hand. To do this, PV angular errors were averaged over 6 consecutive reach training trials with the aligned or rotated cursors. From these average errors, initial and final PV angular errors when reaching with an aligned cursor (Block 1) and rotated cursor (Block 3) were compared using a 3 Group (Instructed vs. Non-Instructed vs. Control) x 2 Cursor (aligned vs. rotated) x 2 Time (mean of first bin of 6 trials vs. last bin of 6 trials) mixed analysis of variance (ANOVA), with repeated measures (RM) on the last two factors. In addition, catch trials were used to confirm reach adaptation. Analyses and results with respect to the catch trials are provided in the S1 File.

Following this, we established if participants were able to adapt to the 40° cursor rotation on Day 2 with the right hand. Similar to Day 1, PV angular errors were averaged over 6 consecutive reach training trials with a rotated cursor. From these average errors, initial (first bin of 6 trials) and final (last bin of 6 trials) PV angular errors when reaching with a rotated cursor (Block 2) were compared using a 3 Group (Instructed vs. Non-Instructed vs. Control) x 2 Time (mean of first bin of 6 trials vs. mean of last bin of 6 trials) mixed ANOVA with RM on the last factor.

Having established that participants adapted their movements, we next examined performance during the PDP (no-cursor) reaching trials. Analyses and results related to RT and MT during PDP trials are provided in the S1 File. To determine explicit and implicit contributions to visuomotor adaptation in the trained and untrained hands, PV angular errors were averaged across targets within each PDP block of inclusion or exclusion trials completed. These average errors for each hand within each block were used to establish explicit and implicit indices according to the following formulas:

$$Explicit\;index\;\left(EI\right)=M_{Inclusion}‐M_{Exclusion}$$

$$Implicit\;index\;\left(II\right)=M_{Exclusion}$$

To determine potential explicit contributions to visuomotor adaptation in both hands and how they changed over time, explicit indices were analyzed using a 2 Group (Instructed vs. Non-Instructed) x 2 Hand (Left vs. Right) x 4 Time (PDP Time 1 vs. PDP Time 2 vs. PDP Time 3 vs. PDP Time 4) mixed ANOVA, with RM on the last two factors. The Control group was excluded from this analysis as they did not perform inclusion trials. Implicit indices were compared across all groups of participants using a 3 Group (Instructed vs. Non-Instructed vs. Control) x 2 Hand x 4 Time mixed ANOVA, with RM on the last two factors. Only 18 PDP reaching trials for the Control group were included in the analysis (either the first 18, or the last 18 trials).

The significance value for all statistical tests was set at p < .05, and Bonferroni post-hoc tests corrected for multiple comparisons were used to find the locus of significant effects for all pre-planned comparisons. Partial eta squared (η_p_^2^) are reported as measures of effect size. All analyses were performed using the statistical software package SPSS 25 for Windows 10.

## Results

### Post-experiment questionnaire

According to responses on the questionnaire, all participants within all 3 groups were classified as aware of the visuomotor distortion. In other words, all participants reported that (i) the cursor did not go where they intended it to go, or ii) they used a strategy (i.e., “pushed” their hand left) in order to successfully land the cursor on the target, when completing the rotated reach training trials with their left (Day 1) and right hand (Day 2). Interestingly, while all participants were designated as aware of the visuomotor distortion based on their verbal responses, it is evident that their knowledge regarding the nature of the visuomotor distortion varied greatly between participants and across groups of participants. Verbal responses in the Non-Instructed and Control groups of participants ranged from “I had to push my hand a little bit to the left (e.g., 10°),” to the “Cursor’s path was rotated CW relative to my hand path by 40°”. As expected, all participants in the Instructed group reported being aware that the cursor was rotated 40° CW relative to their hand motion.

### Reach performance in reach training trials

**Fig 3:**
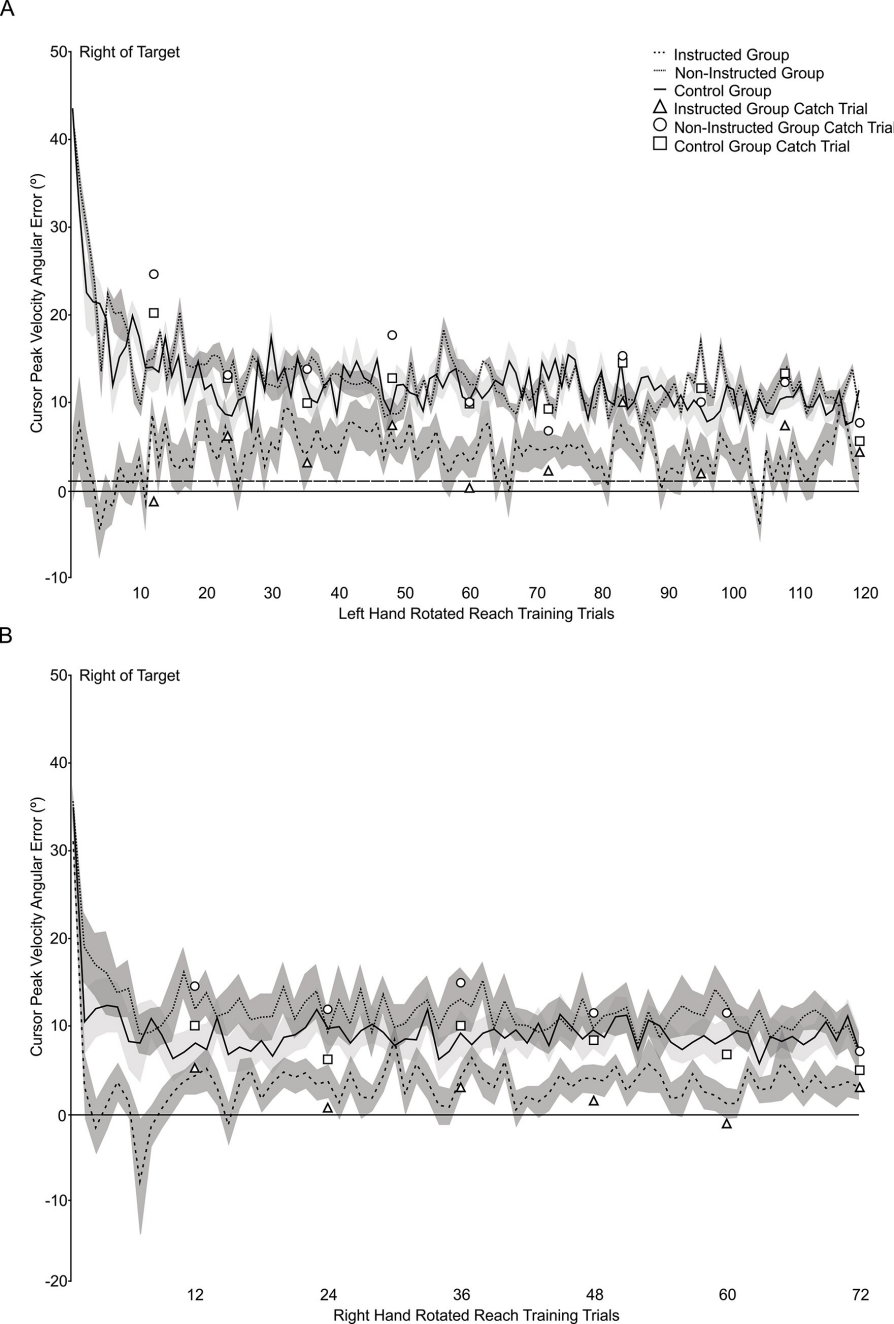
Average reaching errors across rotated reach training trials with the (A) left (trained) and (B) right (untrained) hands. Average cursor directional errors when reaching with the left (A: Block 3, Day 1) and right (B: Block 2, Day 2) hands during rotated reach training. The dashed, dotted, and solid lines represent average reach direction errors for the Instructed, Non-Instructed, and Control groups across rotated reach training trials, respectively. For all groups, the shaded region surrounding each line represents the standard error of the group mean. The triangle, circle, and square symbols represent average errors on catch trials, when no cursor was displayed for the Instructed, Non-Instructed and Control groups, respectively. In (A), the dashed straight line represents the average cursor directional error during aligned reach training trials across all participants. Positive cursor PV angular errors indicate the cursor position was to the right of the target, while negative cursor PV angular errors indicate the cursor position was to the left of the target.

At the end of aligned reach training, all groups reached fairly accurately such that the Instructed group reached with an average cursor PV angular error of 1.72° (SD = 2.16°) to the right of the target, compared to errors of 0.96° (SD = 1.97°) and 1.16° (SD = 2.75°) to the right of the target for the Non-Instructed and Control groups, respectively. Fig 3A presents mean cursor PV angular errors relative to the target over reach training trials with the 40° visuomotor distortion for participants in the Instructed group (dashed line), Non-Instructed group (dotted line), and Control group (solid line) with the left hand on Day 1. From this Figure, it is evident that while all participants adapted their reaches to the visuomotor distortion, how quickly reaches were adapted and the overall extent of reach adaptation achieved were dependent on the group participants were assigned to. In accordance with this suggestion, ANOVA revealed a Group x Cursor x Time interaction [F(2,56) = 30.916, p *<* 0.001, η_p_^2^ = 0.525]. Post hoc analyses indicated that the Instructed group showed greater reach adaptation than the Non-Instructed group when first introduced to the visuomotor distortion (p < 0.001; cursor PV angular errors: Instructed group M = 0.72° (SD = 10.34°) right of the target vs. Non-Instructed group M = 26.47° (SD = 8.66°) right of the target), and at the end of the reach training trials (p = 0.023; Instructed Group M = 4.22° (SD = 6.39°) vs. Non-Instructed Group M = 10.21° (SD = 5.62°)). Similarly, the Instructed group showed greater reach adaptation than the Control group at the start of rotated reach training (p < 0.001; Control Group M = 25.86° (SD = 4.58°)). At the end of training, the Control group (M = 8.55° (SD = 6.46°)) did not differ significantly from the Instructed (p = 0.153) or Non-Instructed groups (p = 1.0).

Right hand reach training performance on Day 2 with the rotated cursor is shown in Fig 3B. Initial cursor PV angular errors were 5.27° (SD = 7.47°), 19.07° (SD = 12.16°), and 14.43° (SD = 8.62°) to the right of the target for the Instructed, Non-Instructed and Control groups, respectively. While, the Instructed group was not reminded of the reaching strategy at this time, they still demonstrated smaller initial (i.e., 1^st^ bin) cursor PV angular errors compared to the Non-Instructed and Control groups (Group x Time interaction [F(2,56) = 5.024, p = 0.010, η_p_^2^ = 0.152], (both p < 0.014)). This trend was also evident at the end of rotated reach training (i.e., last bin; Instructed group M = 1.92° (SD = 4.84°) vs. Non-Instructed group M = 9.29° (SD = 7.22°); p = 0.003 and Instructed group vs. Control group M = 8.07° (SD = 7.53°); p = 0.017)). The Non-Instructed and Control groups did not differ from each other at the start or end of reach training (both p > 0.404). Thus, reach training trials indicate that the Instructed group adapted their reaches earlier and to a greater extent during the rotated reach training trials compared to the Non-Instructed and Control groups when reaching with both the left (trained) and right (untrained) hands.

#### PDP trials: Explicit and implicit adaptation

**Fig 4:**
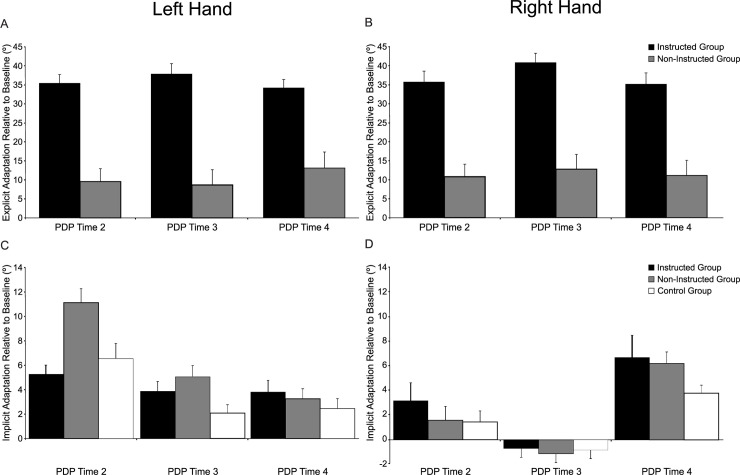
Magnitude of explicit and implicit adaptation during PDP trials. (A-B): Magnitude of explicit adaptation achieved in the left (A) and right (B) hands at PDP Time 2, PDP Time 3, and PDP Time 4 relative to baseline (i.e., PDP Time 1). Black and grey bars represent the magnitude of explicit adaptation for the Instructed and Non-Instructed groups, respectively. (C-D): Magnitude of implicit adaptation achieved in the left (C) and right (D) hands at PDP Time 2, PDP Time 3, and PDP Time 4 relative to baseline (i.e., PDP Time 1). Black, grey, and white bars represent the magnitude of implicit adaptation for the Instructed, Non-Instructed, and Control groups, respectively. Error bars reflect standard error of the mean.

In order to demonstrate explicit and implicit adaptation, the magnitudes of explicit and implicit indices relative to baseline (i.e., PDP Time 1) are shown in Fig 4; note that data for all 4 PDP blocks included in the corresponding analysis are shown in the (S1 File and S1 Fig). As seen in Fig 4, the magnitude of explicit adaptation consistently differed across groups in PDP Blocks 2, 3 and 4. Analysis of EIs revealed a significant Group x Time interaction [F(3,111) = 22.345, p < 0.001, η_p_^2^ = 0.377]. Both the Instructed and Non-Instructed groups showed similar EIs at PDP Time 1 (M = 0.83°, SD = 3.52°; p = 0.731), and significant changes in EIs at PDP Times 2, 3, and 4 compared to PDP Time 1 across the left and right hands (all p < 0.024).

As expected, the Instructed group had greater explicit adaptation than the Non-Instructed group at PDP Times 2, 3 and 4 when reaching with both the left and right hands (all p < 0.001). ANOVA also revealed a Time x Hand interaction [F(3,111) = 4.814, p = 0.003, η_p_^2^ = 0.115]. Post hoc analyses indicated that explicit adaptation was maintained at a similar magnitude when reaching with the left hand across PDP Blocks for both the Instructed (M = 36.61°, SD = 10.28°; all p > 0.886) and Non-Instructed groups (M = 10.30°, SD = 18.13°; all p > 0.072). Explicit adaptation was similar when reaching with the right hand at the corresponding PDP Blocks for both groups except for in PDP Block 3, where both groups showed greater explicit adaptation in the right hand compared to the left hand (Instructed group: left hand M = 38.30°, SD = 11.36° vs. right hand M = 41.07°, SD = 12.66°; Non-Instructed group: left hand M = 8.08°, SD = 18.43° vs. right hand M = 13.10°, SD = 19.41°; both p < 0.039).

In comparison to explicit adaptation, and as shown in Fig 4, changes in the magnitude of IIs relative to PDP Time 1 were fairly small (range = 5°– 11° across all groups). Analysis of IIs revealed a significant Group x Time x Hand interaction [F(6, 168) = 3.269, p = 0.005, η_p_^2^ = 0.105]. Performance between the groups at PDP Time 1 was similar for both the left and right hands (all p = 1.0). All groups showed significant changes in IIs when reaching with the left (trained) hand at PDP Time 2, compared to baseline (all p < 0.002). Post-hoc analyses revealed that the Non-Instructed group (M = 11.34°, SD = 6.97°) had greater implicit adaptation when reaching with the left (trained) hand at PDP Time 2, compared to the Instructed (M = 5.35°, SD = 3.86°) and Control groups (M = 6.32°, SD = 6.74°; both p < 0.034), which did not differ from each other (p = 1.0).While the extent of implicit adaptation at PDP Times 3 and 4 in the left hand did not differ between groups (all p = 1.0), retention of implicit adaptation on Day 2 was only significant (i.e., different from PDP Time 1) in the Instructed and Non-Instructed groups (i.e., Instructed and Non-Instructed groups: PDP Times 3 and 4 vs PDP Time 1, all p < 0.022; Control group: PDP Times 3 and 4 vs PDP Time 1, both p > 0.133).

With respect to transfer of implicit adaptation to the right hand, we found that none of the groups showed significant implicit adaptation when reaching with the right hand at PDP Time 2, after training with the left hand, or the following day at PDP Time 3 (all p > 0.168). Implicit adaptation in the right hand was only seen in all groups at PDP Time 4 (all p < 0.022), after completing the rotated reach training trials with their right hand. The magnitude of implicit adaptation was similar between groups at PDP Time 4 (M = 5.49°, SD = 5.77°; all p > 0.516).

Together, these results indicate that while initial explicit and implicit adaptation in the trained hand differed depending on instructions provided, only explicit adaptation was transferred between limbs and retained across time (i.e., for at least 24 hours).

## Discussion

The main objectives of the current study were to determine whether explicit and/or implicit adaptation transfer between limbs and establish if this transfer is dependent on instructions provided regarding changes in reaches required to counteract the visuomotor distortion. We further asked if explicit and implicit adaptation are retained in both the trained and untrained hands for 24 hours following initial reach training. To address our questions we used the process dissociation procedure (PDP) of Werner and colleagues [25] in order to simultaneously measure explicit and implicit adaptation in the trained and untrained hands over time, independent of participants’ reported awareness of the visuomotor distortion. In interpreting our results below, we have assumed that participants were able to engage and disengage from using a cognitive strategy when asked to do so (i.e., in inclusion and exclusion trials respectively), and acknowledge that all participants were aware that a visuomotor distortion had been introduced.

### Explicit and implicit adaptation: Trained hand

In agreement with previous work using the PDP [e.g., 18, 24, 25, 31], our participants showed evidence of both explicit and implicit adaptation in the left hand, following reach training. Moreover, we found that the magnitude of explicit and implicit adaptation differed depending on instructions provided. The Instructed group demonstrated greater explicit adaptation, when expressed in terms of magnitude and as a percentage of reach adaptation achieved, compared to the Non-Instructed group (M = 36° vs. M = 9°, respectively). In contrast, the Non-Instructed group showed greater implicit adaptation (M = 11°), compared to the Instructed group (M = 5°). This data confirms our previous proposal that being provided with instructions on how to counteract a visuomotor distortion tends to decrease implicit adaptation [18, see also 25, 31].

Regardless of group, the magnitude of implicit adaptation was limited across participants, peaking at approximately 11° in the Non-Instructed group. Limited implicit adaptation as assessed within the PDP has recently been demonstrated by Neville and Cressman [18], and Modchalingam et al. [31], who have collectively suggested that implicit adaptation reaches a ceiling level at approximately 15°, regardless of the visuomotor rotation size that participants train with. While we did not manipulate the size of the visuomotor rotation, the current experiment once again demonstrates that implicit adaptation is limited in extent and supplements previous findings by indicating that this limit is present even when training the left (non-dominant) hand. Based on our findings, it seems that the magnitude of implicit adaptation is rigid [32], while explicit adaptation is more flexible, changing greatly in magnitude depending on instructed provided to participants regarding the visuomotor distortion (i.e., if they are made aware of the visuomotor distortion directly or become aware on their own accord).

Surprisingly, we found that the Non-Instructed group showed greater implicit adaptation compared to the Control group (M = 6°). The Control group was included in the current experiment because we hypothesized that simply asking participants to reach using a strategy during the PDP assessment trials may promote and/or magnify explicit adaptation at the expense of implicit adaptation [as suggested by 27, 28]. Given that implicit adaptation did not differ between the Control and Instructed groups, our hypothesis was not supported. Instead, our results suggest that implicit adaptation, as assessed via the PDP, is not affected by having to reach strategically during assessment trials.

### Transfer of explicit and implicit adaptation to the untrained hand

Having established differences in the extent of explicit and implicit adaptation in the trained hand across groups, we looked to establish if either process transferred between limbs. Werner and colleagues [24] have recently shown that the extent of intermanual transfer is directly related to the extent of explicit adaptation observed in the trained hand. Thus, we would expect greater intermanual transfer in our Instructed group. This is what we found. The Instructed group showed the greatest explicit adaptation in the untrained hand. While we did find greater transfer of explicit adaptation to the untrained hand for the Instructed group compared to the Non-Instructed group, both groups transferred the extent of explicit adaptation observed in their trained hand. In other words, the extent of explicit adaptation observed in the trained hand was transferred completely (100%) to the untrained hand, regardless of instructions provided. Based on our results, it is evident that both hands are able to effectively utilize cognitive strategies gained regarding how to move in the novel visuomotor environment.

In contrast to the significant transfer of explicit adaptation, we did not find significant transfer of implicit adaptation to the right hand, across any of our groups of participants (Mean = 3°). Significant implicit adaptation in the right hand was only observed after participants had completed reach training trials with the right hand on Day 2 of testing. Once again, we observed an upper limit to implicit adaptation of approximately 7°, which remains within the range previously shown by our lab [18].

Together, these results indicate that the overall extent of intermanual transfer (i.e., explicit + implicit adaptation observed in the untrained hand), differed between groups, even though all participants were designated as aware of the visuomotor distortion. In accordance with these findings, previous work by Wang et al. [7], has suggested that intermanual transfer does not depend on participants’ awareness of the visuomotor distortion. In contrast to our results demonstrating transfer of explicit adaptation between limbs, Wang et al. [7] showed that intermanual transfer can be driven implicitly. The difference in results across the two paradigms with respect to the intermanual transfer may be due to the manner of assessment, the size of the rotation introduced and/or engagement of a cognitive strategy. Similar to the results obtained in our exclusion trials, Taylor and colleagues [9] also found limited intermanual transfer of implicit adaptation when assessed via aftereffect trials (i.e., trials like our exclusion trials in which a cursor is not presented and participants are instructed to reach so that their hand goes to the target). In the study by Wang and colleagues [7] significant intermanual transfer was observed when participants reached with their untrained hand when was a cursor was presented. Thus, perhaps implicit processes play a greater role in intermanual transfer if participants have the opportunity to see the cursor during transfer (i.e., direct-effects are assessed and not aftereffects; see [11]), as in the task of Wang et al. [7], and implicit contributions are not dissociated from explicit contributions to visuomotor adaptation as in the current study. With respect to the size of the visuomotor distortion, we had participants adapt to a 40° visuomotor distortion, while Wang et al. [7] had participants adapt to a 32° distortion. Previous research has demonstrated that the contributions of a cognitive strategy (i.e., explicit adaptation) to visuomotor adaptation increase with rotation size, while implicit contributions decrease [18]. All our participants in our Instructed and Non-Instructed groups demonstrated the use of a cognitive strategy in the trained and untrained hand as established by significant explicit adaptation, where the extent of this explicit adaptation and hence intermanual transfer, was greatest in our Instructed group. Perhaps the transfer of implicit adaptation observed by Wang and colleagues [7] is driven by limited explicit adaptation experienced in the trained hand, due to the small cursor rotation. Future research is required to determine the contributions of explicit and implicit processes to visuomotor adaptation and intermanual transfer when such factors as methods of assessment (i.e., trials with cursor present or absent and dissociation of explicit vs. implicit adaptation), rotation size, and use of a cognitive strategy are manipulated. For now, we conclude that explicit adaptation in the form of a cognitive strategy seems to be primarily responsible for intermanual transfer, at least when participants adapt to a large cursor rotation (i.e., 40° or greater), that they were aware of.

### Retention of explicit and implicit visuomotor adaptation

We last looked to establish the stability of explicit and implicit adaptation in the trained and untrained hands over time (i.e., following a 24-hour delay). We found that explicit adaptation was retained across a 24-hour period. In fact, we found that 100% of the initial (i.e., PDP Time 2) explicit adaptation observed in the trained left hand was retained 24 hours later. A similar amount of retention was seen in the untrained right hand, suggesting that explicit adaptation is accessible to both hands 24 hours following initial training.

We observed a decay in implicit adaptation in the trained left hand when it was assessed following a 24-hour delay. Previous work from our lab [e.g., 33] has demonstrated a decay in implicit adaptation when assessed via aftereffects after training with a 30° CW visuomotor distortion with the right hand following a 24 hour delay period (i.e., aftereffects decayed from 24° to 7°; see also [34]). As well, using the PDP, Neville & Cressman [18] observed evidence of implicit decay within just 5 minutes. Based on these results, it seems that implicit adaptation is less stable than explicit adaptation, indicating that explicit processes drive the (long-term) retention of visuomotor adaptation.

### Methods of assessment: Explicit and implicit adaptation

Previous literature has, for the most part, indirectly assessed explicit and implicit contributions to visuomotor adaptation, intermanual transfer and retention. Participants were categorized as either aware or unaware of the visuomotor distortion based on responses to a post-experiment questionnaire and/or how a visuomotor cursor distortion was introduced (i.e., gradually vs. abruptly; [7, 9, 15, 35]). Following this classification, reaching errors were assumed to reflect explicit (if participants were classified as aware) or implicit (if participants were classified as unaware) adaptation. The utility of using questionnaires to establish awareness and varying the introduction of cursor rotations to manipulate participants’ awareness of the visuomotor distortion have been shown to be problematic. Verbal reports tend to underestimate participants’ awareness, possibly due to differences in retrieval contexts between motor and verbal responses [36]. As well, verbal reports may not exhaustively establish a participant’s level of awareness, given the insensitivity of verbal assessment methods to establish knowledge held with low confidence [37–40]. In support of this last proposal, Modchalingam and colleagues [31] recently demonstrated that by adding a scoring system to the awareness questionnaire of Benson et al. [15], awareness scores correlated moderately with reaching errors observed during their inclusion trials. No such correlation was observed when the outcome of the questionnaire was dichotomous (i.e., aware vs. unaware). In addition to concerns regarding the use of questionnaires to establish awareness, manipulating the manner in which the cursor rotation is introduced, may not always lead to the desired changes in awareness (e.g., debriefing reports indicate that some participants in the gradual cursor rotation groups in work by [7, 9] were aware that the visuomotor mapping had been altered).

In the current study, all participants were classified as aware of the cursor rotation when reaching with their left and right hands (i.e., on Days 1 and 2 respectively), based on their responses to the post-experiment questionnaire of Benson and colleagues [15]. Thus, given the differences in intermanual transfer observed across our groups of participants (in particular with respect to explicit adaptation), our results reveal that intermanual transfer of visuomotor adaptation is independent of participants’ awareness, as shown previously by [7]. That said, categorizing participants as aware vs. unaware of the visuomotor distortion does not provide insight into the cognitive strategies engaged. In order to directly establish the role of explicit processes (i.e., strategic changes in one’s reaches) in intermanual transfer, independent of verbal reports of awareness, we used the PDP of Werner and colleagues [25]. The PDP has the added advantage that it allows for the concurrent determination of implicit changes in participants’ reaches, that have been shown to contribute to visuomotor adaptation in the trained hand even when explicit processes are engaged [18, 25, 31].

The PDP is just one method recently put forward to examine the relationship between explicit and implicit contributions to visuomotor adaptation. Another method that has received much attention in the literature today is Taylor and colleagues’ [17] prediction based verbal reporting task. In Taylor’s task, participants are required to verbally report the direction they plan to aim to in order to get the cursor to the target prior to reaching to the target. Specifically, a circular array of numerical landmarks surrounds the target, and participants report the number they plan to reach to. The distance from the number reported to the target is assumed to reflect participants’ explicit pre-planned aiming strategy, while the difference in number reported and actual aiming direction reflects implicit adaptation. Using a variation of this verbal reporting framework, Poh and colleagues [22] found complete intermanual transfer of an explicit pre-planned aiming strategy when both the trained and untrained hands moved in the horizontal plane, such that approximately 22°, or 100% of the explicit contributions measured in the trained hand transferred to the untrained hand. In contrast to our current findings, they also found significant transfer of implicit adaptation, though the transfer of implicit adaptation was incomplete, with only 11.5°, or 48% of implicit contributions in the trained hand transferring to the untrained hand. In addition to observing intermanual transfer of explicit aiming strategies, recent work by Morehead et al [19] using Taylor and colleagues’ [17] verbal reporting method has also demonstrated that retention of visuomotor adaptation is driven by the recall of an explicit pre-planned aiming strategy specific to action selection.

In the current study we deliberately defined explicit processes as reflecting the engagement of cognitive strategies in order to counteract a given visuomotor distortion, where these strategies can arise due to instructions provided, or on the participant’s own initiative, and are not dependent on participant’s reported awareness of the visuomotor distortion. Comparing trends in performance across PDP and verbal reporting studies suggest that the two paradigms may assess similar explicit and implicit processes. For example, both methods of assessment have found that explicit processes scale with rotation size, while implicit processes plateau [18, 32]. Implicit processes have also been shown to take time to develop and decay with time across the two paradigms [18, 32]. Finally, results from the current experiment corroborate work employing the verbal reporting framework, demonstrating the explicit processes are transferred completely between limbs [22], and are responsible for retention of visuomotor adaptation [19].

While similar trends in explicit and implicit changes in reaches have been shown across the two paradigms, future research is required to establish if the paradigms are assessing the same underlying processes, specifically with respect to explicit processes (i.e., are strategic changes in reaches available for report prior to moving?). Previous work using the verbal reporting framework has suggested that requiring participants to engage in predictive, strategic aiming promotes explicit adaptation at the expense of implicit adaptation [27, 28], while results from our Control group of participants would indicate that this is not the case when assessing explicit and implicit processes within the PDP. Moreover, Poh and colleagues [22] showed significant intermanual transfer of implicit processes that was not observed in our study. Interestingly, the magnitude of implicit adaptation observed by Poh and colleagues [22] in the trained hand is considerably larger than that observed within the current study and falls outside the maximum magnitude of implicit adaptation previously reported [18, 32]. Future research is required to determine if these differences in transfer of implicit adaptation across the paradigms is due to the manner of assessment (i.e., PDP vs. verbal reporting framework), initial magnitude of implicit adaptation in the trained hand and/or methodological differences with regards to the placement of assessment trials within the experimental protocol, as discussed below.

### Reach training paradigm: Visuomotor adaptation and intermanual transfer

Given our interest in examining changes in movement planning in the untrained limb (i.e., errors at peak velocity), we chose to have participants initially train with their non-dominant (left) hand [2, 5, 6]. Participants completed a block of reach training trials with the left hand, after which intermanual transfer was assessed. While all participants adapted their reaches to the visuomotor distortion when completing the reach training trials with their left hand, participants in the Instructed group adapted their reaches earlier and to a greater extent. That is, at the end of the 120 rotated reach training trials on Day 1 and again, when they reached with the right hand on Day 2, the Instructed group demonstrated reduced errors compared to the Non-Instructed and Control groups. These results are contradictory to previous literature, which has shown similar performance by Instructed and Non-Instructed groups by the end of a block of reach training trials [7, 15]. In fact, work from our own lab found similar errors between Instructed and Non- Instructed groups of participants within 30 to 60 reach training trials when reaching with the right (dominant) hand while seeing a cursor that was rotated 40° CW relative to hand motion [18]. It remains to be determined why initially training the left hand, in the absence of instructions, leads to greater reaching errors than seen when initially training the right hand.

In the present study, intermanual transfer was assessed at the end of the reach training blocks. In contrast, Poh and colleagues [22] intermixed reaching trials with the trained and untrained hand, such that visuomotor adaptation was assessed repeatedly (9x) in the untrained hand, following every 20 rotated reach training trials. Using a similar block design to the current study, early work by Taylor and colleagues [9] also showed minimal intermanual transfer of aftereffects (reflecting implicit adaptation). Specifically, they observed that the untrained hand reached with errors of approximately 3° following reach training trials with a cursor that was rotated 22.5°. Taken together, these findings suggest that implicit adaptation does not appear to significantly transfer between hands, regardless of the cursor rotation size (e.g., smaller as in [9], or larger as in the current study) when using a block design. Instead, for implicit adaptation to significantly contribute to intermanual transfer, the untrained hand may need to be engaged early in training. In accordance with this suggestion, Wang and colleagues [23, 41] have argued that it is the absence of reaching movements (active or passive) with the untrained hand during reach training that is responsible for the lack of intermanual transfer. They show that merely having the untrained limb move in the required adapted trajectory (in the absence of visual feedback) during opposite limb training promotes intermanual transfer. Based on the current results, and results from Poh and colleagues [22], explicit adaptation can be transferred to the opposite limb, regardless of experimental design (i.e., blocked versus intermixed trials), and hence whether or not the opposite (untrained) hand is engaged early on during reach training trials.

## Conclusion

Using the PDP framework, we established that intermanual transfer of visuomotor adaptation is primarily driven by explicit processes (i.e., a cognitive strategy), regardless of whether participants are provided with instructions or not on how to counteract the visuomotor rotation. Explicit adaptation observed initially in the trained hand was transferred completely to the untrained (right) hand. In contrast, implicit contributions to visuomotor adaptation did not significantly transfer between hands. Explicit adaptation was also retained in both hands over testing days, while implicit adaptation decayed. Together, these findings suggest that explicit processes are primarily responsible for intermanual transfer and the retention of visuomotor adaptation in both hands when participants adapt to a large visuomotor distortion that they are aware of.

## Supporting information

S1 File Additional data analyses and results. (DOCX)

S1 Fig Reaching errors during PDP trials. (A-B): Reaching errors on Inclusion Trials (Explicit + Implicit Indices) achieved in the left (A) and right (B) hands at PDP Time 1, PDP Time 2, PDP Time 3, and PDP Time 4. Black and grey bars represent the magnitude of Explicit + Implicit Indices for the Instructed and Non-Instructed groups, respectively. (C-D): Reaching errors on Exclusion Trials (Implicit Indices) achieved in the left (C) and right (D) hands at PDP Time 1, PDP Time 2, PDP Time 3, and PDP Time 4. Black, grey, and white bars represent the magnitude of Implicit Indices for the Instructed, Non-Instructed, and Control groups, respectively. (E-F): Explicit Indices (Errors on Inclusion Trials–Errors on Exclusion Trials) achieved in the left (E) and right (R) hands at PDP Time 1, PDP Time 2, PDP Time 3, and PDP Time 4. Black and grey bars represent the magnitude of Explicit Indices for the Instructed and Non-Instructed groups, respectively. Positive values reflect reaching errors to the left of the target. Error bars reflect standard error of the mean.
(EPS)

S2 Fig Percentage of explicit + implicit adaptation relative to reach adaptation. (A-B): Explicit adaptation (white bars) + implicit adaptation (grey bars) in the left (A) and right (B) hands at PDP Time 2, PDP Time 3, and PDP Time 4 expressed relative to the magnitude of reach adaptation achieved in the left or right hands during rotated reach training trials for the Instructed (I), Non-Instructed (N-I), and Control (C) groups. Only implicit adaptation, expressed as a percentage of reach adaptation, is shown for the Control group.
(EPS)
