# Supplementary material for: Intermanual transfer and retention of visuomotor adaptation to a large visuomotor distortion are driven by explicit processes
Source: PLoS One. 2021 Jan 11;16(1):e0245184. doi: 10.1371/journal.pone.0245184 (PMC7799748; doi:10.1371/journal.pone.0245184)
Supplement: S1 File — (DOCX) [file pone.0245184.s001.docx]

S1 File. Intermanual transfer and retention of visuomotor adaptation to a large visuomotor distortion are driven by explicit processes

Jean-Michel Bouchard^1^ & Erin K. Cressman^1^

^1^School of Human Kinetics, University of Ottawa, Ottawa, Canada

**Data Analyses and Results**

**Reach Training Blocks**

**Catch Trials**

Catch trials were used to confirm reach adaptation during the reach training trials. Given the limited number of catch trials completed in each reach training block, PV angular errors on catch trials were averaged over 2 no cursor trials rather than 6 trials like in the reach training blocks. For the left hand, PV angular errors in the catch trials were compared using a 3 Group (Instructed vs. Non-Instructed vs. Control) x 3 Time (mean of first bin of 2 aligned catch trials vs. mean of first bin of 2 rotated catch trials vs. mean of last bin of 2 rotated catch trials) mixed analysis of variance (ANOVA), with repeated measures (RM) on the last factor. For the right hand, there was no block of aligned reach training trials completed, thus, errors on catch trials completed in the right hand rotated reach training block were compared in a 3 Group (Instructed vs. Non-Instructed vs. Control) x 2 Time (mean of first bin of 2 rotated catch trials vs. mean of last bin of 2 rotated catch trials) mixed ANOVA, with RM on the last factor.

Analyses of catch trials completed with the left hand on Day 1 showed similar findings to the reach training trials (see Figure 3), specifically a Group x Time interaction [F(4,112) = 6.70, p < 0.001, η_p_^2^ = 0.193]. All groups had similar errors during the catch trials in the aligned reach training block (all p > 0.058). The Instructed group reached with similar errors in both bins of catch trials performed during rotated reach training (p = 0.376), but the Non-Instructed and Control groups showed a significant decrease in cursor PV angular errors over the course of the reach training trials (both p < 0.004). These results imply that the Instructed group changed their reaches immediately when reaching with the visuomotor distortion, but participants in the Non-Instructed and Control groups took longer to adapt their reaches.

With respect to right hand performance, analyses of the catch trials revealed a significant main effect of Group [F(2,56) = 5.549, p = 0.006, η_p_^2^ = 0.165], with post hoc analysis revealing that the Instructed group reached with less error at PV compared to the Non-Instructed group (p = 0.005). PV angular errors in the Control group did not differ statistically from either group (both p > 0.269). Together, results from the catch trials complement the findings from the reach training trials, indicating that the Instructed group adapted their reaches earlier and to a greater extent during the rotated reach training trials compared to the Non-Instructed and Control groups when reaching with both the left (trained) and right (untrained) hands.

**PDP Blocks**

**Reaction Time (RT) and Movement Time (MT)**

RT and MT during PDP exclusion trials were both assessed using a 3 Group (Instructed vs. Non-Instructed vs. Control) x 2 Hand (trained (left) vs untrained (right)) x 4 Time (PDP Time 1 (Day 1, Block 2) vs. PDP Time 2 (Day 1, Block 4) vs. PDP Time 3 (Day 2, Block 1) vs. PDP Time 4 (Day 2, Block 3)) mixed ANOVA, with RM on the last two factors. For the Control group, only data from 18 PDP reaching trials were included in the analysis (corresponding to the 18 exclusion trials performed by the other 2 groups of participants). For half of the participants in the Control group, these 18 PDP trials corresponded to the first set of 18 PDP trials completed. For the other Control participants, the second set of 18 PDP trials were used. RT and MT during PDP inclusion trials were assessed using a 2 Group (Instructed vs. Non-Instructed) x 2 Hand x 4 Time mixed ANOVA, with RM on the last two factors. The Control group was excluded from this analysis, as those assigned to this group did not perform inclusion trials. These analyses were performed for both inclusion and exclusion trials individually to establish whether instructions were linked to changes in RT or MT. RT and MT variability during inclusion and exclusion trials were analyzed in a similar manner. Finally, paired samples t-tests were used to determine whether mean RT and/or MT differed between inclusion and exclusion trials, for the Instructed and Non-Instructed groups.

Overall, RT was 402.78 ms across all exclusion trials. Analysis of RT on exclusion trials revealed an effect of Hand [F(1,56) = 5.753, p = 0.020, η_p_^2^ = 0.093], such that RT was longer when reaching with the right hand (M = 410.64 ms, SD = 101.13 ms) compared to the left hand (M = 394.92 ms, SD = 82.12 ms). Otherwise, RT did not vary significantly across Group or Time (both p > 0.138). RT variability was on average 88.86 ms (SD = 71.62 ms), and did not differ significantly across Group, Hand or Time (all p > 0.293).

RT for inclusion trials tended to be longer than exclusion trials, such that it was 441.92 ms on average across all PDP inclusion trials. In support of this observation, t-tests revealed that average RT on inclusion PDP trials across PDP Times 2, 3, and 4, with the left hand, was significantly longer for the Instructed and Non-Instructed groups compared to average RT during exclusion PDP trials across the same PDP trials (both p < 0.001). Moreover, RT was longer for right hand inclusion trials compared to right hand exclusion trials for the Instructed group (p < 0.001). There was no difference in RT between the right hand inclusion and exclusion trials completed by the Non-Instructed group (p = 0.102).

Comparing RT on inclusion trials across groups, ANOVA revealed a significant Group x Time interaction [F(3,111) = 7.027, p < 0.001, η_p_^2^ = 0.160]. There was no difference between the 2 groups at PDP Time 1, following aligned reach training (M = 392.93 ms, SD = 78.67 ms; p = 0.563). However, the Instructed group had significantly longer RT on inclusion trials completed at PDP Times 2, 3 and 4 compared to PDP Time 1 (all p < 0.001), and RT at PDP Times 2, 3 and 4 did not differ from each other (M = 496.74 ms, SD = 89.64 ms; all p = 1.0). Moreover, the Instructed group had significantly longer RT on inclusion trials compared to the Non-Instructed group at PDP Times 3 and 4 (M = 407.80 ms, SD = 97.18 ms; both p < 0.007), and there was a trend for longer RT at PDP Time 2 (M = 449.43 ms, SD = 112.11 ms; p = 0.083). Analyses of RT variability also revealed a significant Group x Time interaction [F(3,111) = 3.322, p = 0.022, η_p_^2^ = 0.082]. RT variability for the Non-Instructed group remained consistent across all PDP Times (M = 97.93 ms, SD = 67.90 ms; all p > 0.181), but the Instructed group showed greater RT variability at PDP Times 3 and 4 on Day 2 of testing (M = 111.67 ms, SD = 60.39 ms) compared to PDP Time 1 (M = 71.75 ms, SD = 34.68 ms; both p < 0.022). In addition, the Instructed group demonstrated greater RT variability than the Non-Instructed group at PDP Time 3 (p = 0.038).

MT was on average 943.39 ms during exclusion trials. MT did not significantly differ across Group or Time, but ANOVA revealed a main effect of Hand [F(1,56) = 9.853, p = 0.003, η_p_^2^ = 0.150], such that reaches with the trained (left) hand (M = 958.34 ms, SD = 262.81 ms) had a longer MT than reaches with the untrained (right) hand (M = 928.45 ms, SD = 263.09 ms). With respect to analysis of MT variability during exclusion PDP trials, ANOVA revealed a Group x Time x Hand interaction [F(6,168) = 2.471, p = 0.026, η_p_^2^ = 0.081] and post hoc analyses indicated that the Instructed group showed greater MT variability when reaching with the left hand during PDP Trials at Time 2 compared to the Non-Instructed and Control groups (both p < 0.047). In contrast, no other differences were found between the groups at any other time when using the left hand (all p > 0.912). The Non-Instructed group also showed greater MT variability when reaching with the right hand during PDP Times 1 and 2 compared to the Control group (both p < 0.029), but the groups showed similar MT variability at all other PDP Times (all p > 0.133).

MT for inclusion trials was longer than exclusion trials, such that is was 1019.30 ms on average across all Groups, Hands and Time. As expected, t-tests revealed that MT for both hands for the Instructed and Non-Instructed groups was longer in the inclusion PDP trials compared to exclusion PDP trials (all p < 0.018). Compared to exclusion trials, ANOVA revealed no significant changes in MT during inclusion trials across Group, Hand or Time (all p > 0.085). MT variability during inclusion trials was on average 152.32 ms, which also did not differ significantly across Group, Hand or Time (all p > 0.057).

Together, these RT and MT results indicate that, in general, all groups performed with similar RT and MT on exclusion trials. Participants in the Instructed and Non-Instructed groups then increased their preparation time (i.e., RT) and the time it took to execute a movement (i.e., MT) when reaching with both hands on inclusion trials compared to exclusion trials. Furthermore, participants in the Instructed group increased their preparation time even more on inclusion trials compared to participants in the Non-Instructed group after training with a rotated cursor.

**Reaching Errors**

**Errors during Inclusion and Exclusion Trials**

##### **S1 Fig. Reaching Errors during PDP trials.** (A-B): Reaching errors on Inclusion Trials (Explicit + Implicit Indices) achieved in the left (A) and right (B) hands at PDP Time 1, PDP Time 2, PDP Time 3, and PDP Time 4. Black and grey bars represent the magnitude of Explicit + Implicit Indices for the Instructed and Non-Instructed groups, respectively. (C-D): Reaching errors on Exclusion Trials (Implicit Indices) achieved in the left (C) and right (D) hands at PDP Time 1, PDP Time 2, PDP Time 3, and PDP Time 4. Black, grey, and white bars represent the magnitude of Implicit Indices for the Instructed, Non-Instructed, and Control groups, respectively. (E-F): Explicit Indices (Errors on Inclusion Trials – Errors on Exclusion Trials) achieved in the left (E) and right (R) hands at PDP Time 1, PDP Time 2, PDP Time 3, and PDP Time 4. Black and grey bars represent the magnitude of Explicit Indices for the Instructed and Non-Instructed groups, respectively. Positive values reflect reaching errors to the left of the target. Error bars reflect standard error of the mean.

**Explicit + Implicit Adaptation**

We compared the overall extent of explicit + implicit adaptation in PDP trials expressed as a percentage of reach adaptation observed in the corresponding reach training blocks between the Instructed and Non-Instructed groups in a 2 Group x 2 Hand x 3 Time (PDP Time 2 (Day 1, Block 4) vs. PDP Time 3 (Day 2, Block 1) vs. PDP Time 4 (Day, Block 3)) mixed ANOVA, with RM on the last two factors. The data was log transformed to ensure a normal distribution. Explicit and implicit adaptation were defined in PDP Blocks 2, 3 and 4 by subtracting the EI or II at PDP Time 1, respectively. The extent of reach adaptation corresponding to PDP Blocks 2 and 3 was considered to be the mean PV angular error of the last 6 rotated reach training trials with the left hand on Day 1 (i.e., Block 3, Day 1) minus the mean PV angular error of the last 6 aligned reach training trials with the left hand on Day 1 (i.e., Block 1, Day 1), while the extent of reach adaptation corresponding to PDP Block 4 was considered to be the mean PV angular error of the last 6 rotated reach training trials with the right hand on Day 2 (i.e., Block 2, Day 2) minus the mean PV angular error at PDP Time 1 when reaching with the right hand (i.e., Block 2, Day 1). The Control group was excluded from these analyses as they did not perform inclusion trials.

The sums of explicit + implicit adaptation achieved during PDP trials as a percentage of reach adaptation are displayed below (Supplementary Figure 2), with implicit adaptation in the Control group expressed as a percentage of reach adaptation included as well. A Group x Hand x Time interaction [F(2,60) = 7.526, p < 0.001, η_p_^2^ = 0.201], revealed that explicit + implicit adaptation in the Instructed group during PDP trials was similar to the extent of reach adaptation observed in both hands when reaching with the cursor during rotated reach training trials (Explicit M = 103.59%, SD = 25.07%; Implicit M = 15.34%, SD = 11.93%; Explicit + Implicit M = 118.93%, SD = 24.56%). This percentage of reach adaptation accounted for by explicit + implicit adaptation did not differ across Hand or Time for the Instructed group (all p > 0.428). In contrast, the percentage of reach adaptation accounted for by explicit + implicit adaptation in the Non-Instructed group was less than the Instructed group at all PDP times in both hands (all p < 0.004). Moreover, greater reach adaptation in the Non-Instructed group was accounted for by explicit + implicit adaptation in the trained (left) hand at PDP Time 2 (Explicit M = 24.89%, SD = 50.90%; Implicit M = 40.33%, SD = 26.74%; Explicit + Implicit M = 65.22%, SD = 38.80%) compared to PDP Time 3 (Explicit M = 23.12%, SD = 57.82%; Implicit M = 18.52%, SD = 18.85%; Explicit + Implicit M = 41.64%, SD = 50.21%) and PDP Time 4 (Explicit M = 30.76%, SD = 79.17%; Implicit M = 15.08%, SD = 27.91%; Explicit + Implicit M = 45.85%, SD = 58.93%; both p < 0.008), suggesting that significant decay occurred across testing days. Explicit + implicit adaptation for the Non-Instructed group in the right hand did not differ significantly between PDP Time 2 (Explicit M = 32.10%, SD = 52.26%; Implicit M = 5.14%, SD = 26.71%; Explicit + Implicit M = 37.24%, SD = 47.82%) and PDP Time 3 (Explicit M = 40.01%, SD = 58.88; Implicit M = -5.29%, SD = 24.55%; Explicit + Implicit M = 34.72%, SD = 60.14%; %; p = 0.335), but significantly increased following rotated reach training with the same (right) hand (i.e., PDP Time 4 Explicit: M = 26.62%, SD = 67.35%; Implicit M = 20.58%, SD = 25.42%; Explicit + Implicit M = 47.20%, SD = 69.56%; p < 0.018). Together these results indicate that explicit and implicit adaptation accounted for a greater percentage of reach adaptation in the Instructed group compared to the Non-Instructed group. Moreover, explicit and implicit adaptation accounted for a greater amount of reach adaptation following training of a given limb.

##### **S2 Fig. Percentage of explicit + implicit adaptation relative to reach adaptation.** (A-B): Explicit adaptation (white bars) + implicit adaptation (grey bars) in the left (A) and right (B) hands at PDP Time 2, PDP Time 3, and PDP Time 4 expressed relative to the magnitude of reach adaptation achieved in the left or right hands during rotated reach training trials for the Instructed (I), Non-Instructed (N-I), and Control (C) groups. Only implicit adaptation, expressed as a percentage of reach adaptation, is shown for the Control group.
